# Transgenic Tarantula Toxin: A novel tool to study mechanosensitive ion channels in *Drosophila*

**DOI:** 10.1016/j.jinsphys.2020.104116

**Published:** 2020

**Authors:** Denis Beqja, Sunna Haidar, Mihail Nikolaev, Yilan Shen, Barry Denholm

**Affiliations:** Biomedical Sciences, University of Edinburgh, Edinburgh EH8 9XD, UK

## Abstract

The tarantula venom toxin GsMTx4 is the only known specific inhibitor of cation-selective mechanosensitive ion channels (MSCs). Its specificity, potency, and ease of use on isolated tissues and cells have made it a powerful pharmacological tool to identify and probe the physiological function of MSCs. In some contexts, however, it would be desirable to deliver the toxin in a controlled way *in vivo*. Here we describe a novel tool to allow spatial and temporal control of GsMTx4 delivery *in vivo* in *Drosophila*. To test the tool, we targeted MSCs required for mechanical nociception in a specific subset of sensory neurons in intact larvae. Expression of GsMTx4 in these neurons results in robust inhibition of mechanical nociception, demonstrating the toxin is active when expressed *in vivo*. The tool will be particularly useful to manipulate MSC activity in a spatially and temporally-controlled manner to study their role in development, physiology and behaviour in intact, free moving animals.

## Highlights

• A novel genetically-encoded tool to inhibit mechanotransduction in *Drosophila.*
• Robust inhibition of mechanical nociception in larval sensory neurons.
• A powerful tool to study mechanotransduction in intact, free moving animals.

## Graphical abstract

**Figure:**
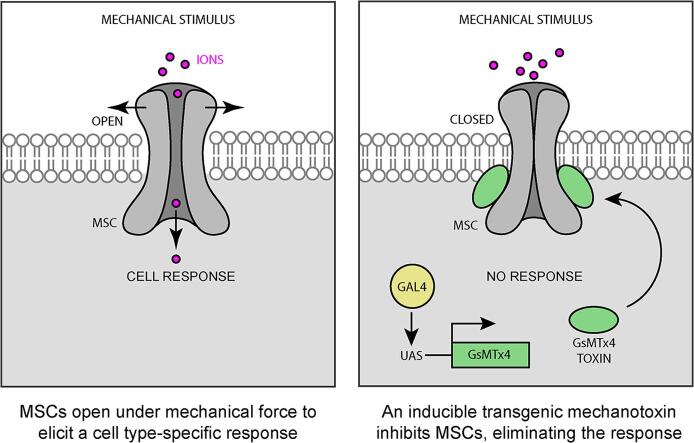

## 1 Introduction

Venom from the tarantula *Grammostola spatulata* contains a potent and specific inhibitor of mechanosensitive ion channels (MSCs). The active agent is a short 34 amino acid peptide called *Grammostola spatulata* mechanotoxin #4 (GsMTx4)(Suchyna, et al., 2000). It is the only known toxin specific for cation-selective MSCs, with known inhibitory activities for the channels of the Piezo and Transient Receptor Potential (TRP) families (Bae et al., 2011, Spassova et al., 2006, Alcaino et al., 2017). It has become an important tool to identify and characterise the physiological roles of MSCs (Bowman et al., 2007, Suchyna, 2017).

GsMTx4 is commercially available and is easily applied to *ex vivo* preparations adding to the power of the toxin as a tool. However, there are some contexts where it would be desirable to deliver the toxin in a non-invasive way. For example, when studying development, integrated physiological systems or behaviour. Furthermore, for some studies it would be advantageous to deliver the toxin to specific organs, tissues or cells. This would be difficult or impossible when the only method for delivery is exogenous application. For these reasons we generated transgenic fly lines with a genetically encoded GsMTx4 gene under the control of the UAS-GAL4 system to allow spatial and temporal delivery of the toxin in the intact animal. Here we describe these lines and assess their ability to inhibit MSCs in the context of mechanical nociception in the larval peripheral nervous system.

## 2 Results & discussion:

**Fig. 1:**
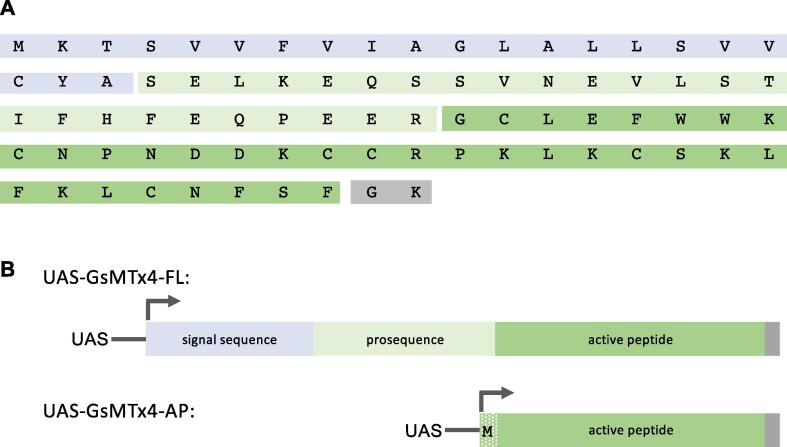
(A) Peptide sequence of GsMTx4. The first 21 amino acids are removed as a signal sequence (blue). The active peptide (dark green) is produced following removal of the last two amino acids during amidation (grey) and cleavage after arginine 46 (to remove the prosequence, light green). (B) Schematic of the GsMTx4 transgenic constructs. Full length and active peptide sequences of GsMTx4 were synthesised and cloned into the pUASTattB vector downstream to the Upstream Activating Sequence (UAS). A start codon (M) was added to the beginning of the AP sequence. Several independent transgenic lines were generated by integration at the ZH-86Fb *attP* site on the third chromosome. The lines are referred to as UAS-GsMTx4-FL (top) and UAS-GsMTx4-AP (bottom). (For interpretation of the references to colour in this figure legend, the reader is referred to the web version of this article.)

We synthesized the *Grammostola spatulata* gene encoding the GsMTx4 peptide, cloned it downstream to the UAS inducible promoter and generated transgenic fly lines (Fig. 1). Two versions were generated: (i) a full-length variant containing the full *GsMTx4* cDNA (GsMTx4-FL) encoding a pre-pro-peptide including a N-terminal signal peptide and a pro-sequence, both of which are subsequently cleaved to make the active peptide; (ii) a variant consisting of the active peptide alone (GsMTx4-AP). As GsMTx4-AP lacks the signal peptide sequence it will not be secreted, and would restrict MSC inhibition to the cells in which it is expressed.

**Fig. 2:**
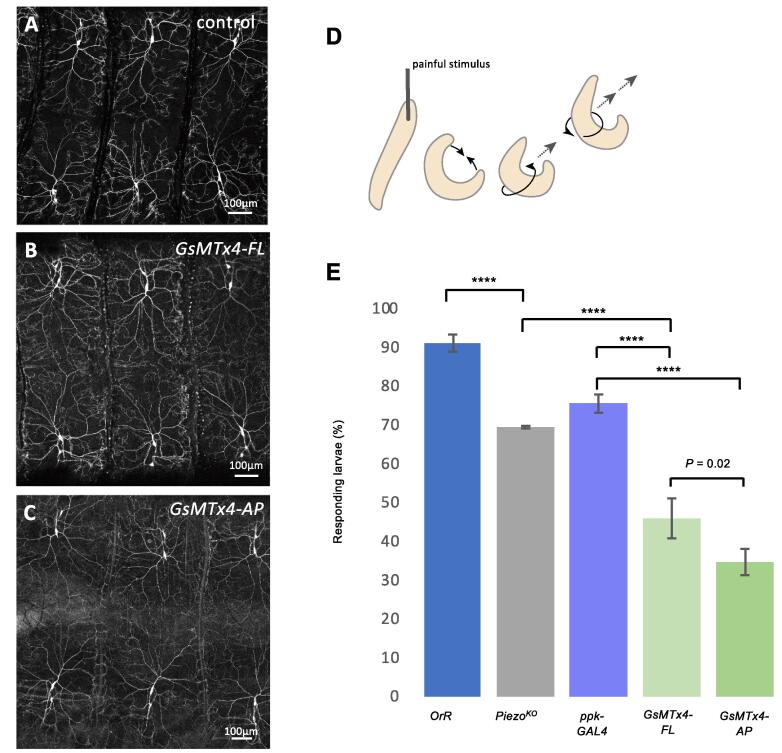
(A) Control third instar larval nociceptive neurons in dorsal abdominal segments 5–7 (*ppk-GAL4 > UAS-CD8-GFP*). (B, C) Third instar larval nociceptive neurons in dorsal abdominal segments 5–7 expressing GsMTx4-FL (B) and GsMTx4-AP (C). (D) Schematic drawing showing typical escape behaviour in response to a painful stimulus: the larva contracts into a crescent then rolls vigorously with corkscrew-like revolutions (black arrows) away from the stimulus (grey arrows). (E) Percentage of larvae responding to a 50 mN mechanical stimulus for *OrR* (blue, *n* = 258), *Piezo^KO^* (grey, *n* = 272), *ppk-GAL4 (purple, n* = 241), *ppk-GAL4 > UAS-GsMTX4-FL* (light green, *n* = 246), *ppk-GAL4 > UAS-GsMTX4-AP* (dark green, *n* = 173). Data show mean ± s.e.m. for at least three independent replicates for each condition, *P* < 0.0001 (****) χ^2^. (For interpretation of the references to colour in this figure legend, the reader is referred to the web version of this article.)

We tested the function of the transgenic GsMTx4 lines by assessing their ability to block mechanical nociception in larval *pickpocket-GAL4* (*ppk-GAL4*) sensory neurons – a subset of multidendritic neurons that tile the body wall and respond to a variety of external stimuli including mechanical force (Fig. 2A) (Zhong et al., 2010). We first checked to determine whether GsMTx4-FL or GsMTx4-AP affect *ppk-GAL4* neuron viability or morphology, and find they do not (Fig. 2B, C). Painful mechanical stimuli are transduced by *ppk* neurons to elicit an escape behaviour in which the larva contracts into a crescent then rolls vigorously with corkscrew-like revolutions away from the stimulus (Fig. 2D and Supplementary Movie 1). We find mechanical stimulation with a 50 mN von Frey filament elicits the escape response in >90% of wild-type 3rd instar larvae (Fig. 2E, Supplementary Movie 1). A number of MSCs, including the Piezo channel, are known to be required in *ppk-GAL4* neurons to transduce nociceptive stimuli; perturbation of any of these channels attenuates escape behavior (Zhong et al., 2010, Kim et al., 2012, Tracey et al., 2003). We used *Piezo^KO^* larvae as a positive control to score for deficits in mechanical nociception. In line with a previous study (Kim et al., 2012), we find a significant proportion (30%) of *Piezo^KO^* larvae do not respond to the 50 mN stimulus (Fig. 2E). Next, we tested the ability of GsMTx4 to block mechanical nociception by driving expression in *ppk-GAL4* neurons. For this experiment we used *ppk-GAL4* heterozygote larvae as controls. We find >75% *ppk-GAL4* heterozygotes respond to the stimulus (Fig. 2E). In contrast, expression of GsMTx4 in *ppk-GAL4* neurons severely attenuates the escape response, with only 46% (GsMTx4-FL) and 35% (GsMTx4-AP) of larvae responding to the stimulus (Fig. 2E, Supplementary Movie 2). These data show that genetically encoded GsMTx4 efficiently blocks mechanical nociception in *ppk-GAL4* neurons when expressed *in vivo*. The phenotype for *GsMTx4-AP* is significantly stronger than *GsMTx4-FL* (*P* = 0.02, Fig. 2E). As *GsMTx4-AP* is designed not to be secreted it is likely the toxin reaches higher concentration within the neurons, possibly explaining its greater potency. We find expression of GsMTx4 (*FL* and *AP*) in *ppk-GAL4* neurons does not result in any other obvious physiological or behavioural defects in either larvae, pupae or adults. In future experiments, it will be interesting to assess the effects of *in vivo* expression of GsMTx4 in other contexts where MSCs have been implicated, for example for epithelial morphogenesis in tissues during embryonic dorsal closure (Hunter et al., 2014), or for stretch-activated mechanotransduction in bipolar dendritic sensory neurons (Suslak, et al., 2015).

Supplementary Movie 1
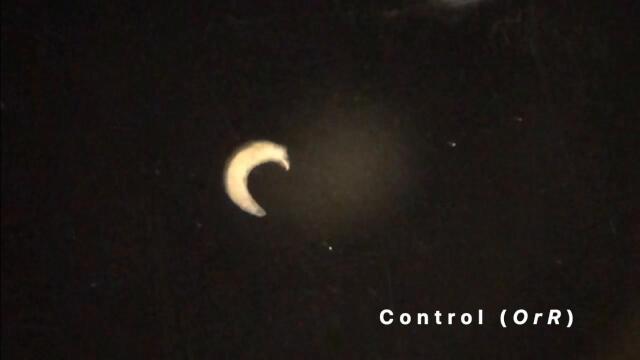

Supplementary Movie 2
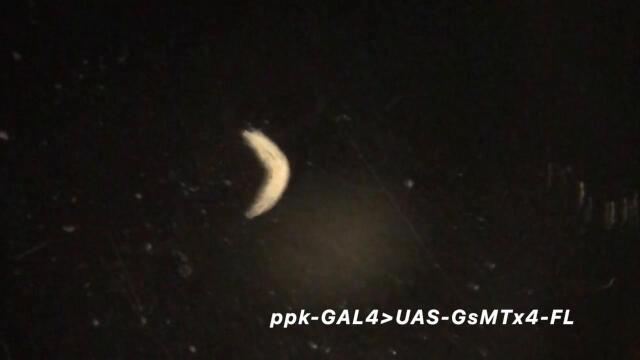

Our results show the genetically encoded GsMTx4 efficiently blocks mechanical nociception in *ppk-GAL4* neurons and strongly imply that its potency as an inhibitor of MSCs is maintained when expressed *in vivo*. A further implication of our findings is that the toxin is active from the intracellular face of the membrane in line with some, but not all, previous studies (Bae et al., 2011, Nishizawa and Nishizawa, 2007, Kamaraju et al., 2010). An important question that remains is which channel(s) are targeted by GsMTx4? Our data show *ppk-GAL4* > GsMTx4 (*FL* and *AP*) produce stronger phenotypes than *Piezo^KO^* (*P ≥* 0.0001), suggesting GsMTx4 inhibits other channels. Three MSCs (Piezo, Painless and Ppk) are expressed in *ppk-GAL4* neurons and are required for mechanical nociception (Zhong et al., 2010, Kim et al., 2012, Tracey et al., 2003). It is conceivable the GsMTx4 phenotype is a combined phenotype resulting from inhibition of all three channels. There is strong evidence for GsMTx4 inhibition of channels from Piezo (although the site of inhibitory action appears to be the extracellular face of the membrane) and TRP (Painless is a TRP channel) families (Bae et al., 2011, Spassova et al., 2006, Alcaino et al., 2017). For this reason, we suggest Piezo and Painless are possible targets of GsMTx4 in *ppk-GAL4* neurons. However, the phenotype observed with GsMTx4 cannot be explained simply by the combined inhibition of Piezo and Painless (even if both are targeted) because genetic data indicate they are part of the same pathway (the phenotype of the double homozygote is no stronger than the homozygote phenotype of either mutation alone) (Kim et al., 2012). It is therefore necessary to postulate additional targets. Pickpocket (an ENac/DEG channel) is a candidate given that it is present and required in *ppk-GAL4* neurons for mechanical nociception, although there is no evidence yet to indicate that GsMTx4 is able to inhibit ENac/DEG channels. A further candidate is the recently described Piezo-like channel (Hu et al., 2019), however it is not known whether *Piezo-like* is required for mechanical nociception or if it is expressed in *ppk-GAL4* neurons. GsMTx4 has also been shown to influence the activity of the TREK mechano-gated potassium channels (Gottlieb and Sachs, 2009, Gnanasambandam et al., 2017). Therefore, it is possible that the loss of mechanical nociception reported here could be accounted for by the activation of the *Drosophila* TREK channel (Sandman) as well as inhibition of MSCs.

It is conceivable that GsMTx4 inhibits multiple different classes of MSCs. Supporting this, there is strong evidence that GsMTx4 exerts its inhibitory effects indirectly by modulating local mechanical tension in the vicinity of the channel upon insertion between membrane lipids, rather than through direct interaction with a specific channel (Suchyna et al., 2004). Future studies combining genetic mutations, GsMTx4 expression and electrophysiological recordings would resolve the identify of MSCs targeted by GsMTx4.

The use of a genetically-encoded tetanus toxin light chain in *Drosophila* has proved to be a powerful tool to identify physiological functions of neurons and to map neuronal circuits (Sweeney et al., 1995). Likewise, we suggest the lines described here will provide a powerful new tool to study MSCs and mechanotransduction *in vivo*. They allow the delivery of the toxin to specific organs, tissues or cells, and can be adapted to provide temporal control of expression simply by crossing into appropriate genetic backgrounds (McGuire et al., 2004). The lines will be particularly useful for studying mechanotransduction during development, physiology and behaviour in intact, free moving animals.

## 3 Materials & methods

### 3.1 Generation of GsMTx4

The *Grammostola spatulata* GsMTx4 gene was codon-optimised for *Drosophila* (OptimumGene™), synthesised, cloned into *Eco*RI-*Xho*I sites in pUASTattB (GenScript) and subsequently transformed into the ZH-86Fb *attP* site on the third chromosome (FlyC31 system) (BestGene Inc.).

### 3.2 Nociception assay

Mechanical nociception was tested as described previously using a calibrated von Frey filament (Kim et al., 2012). Briefly, wandering 3rd instar larvae were transferred into a petri dish with shallow water and allowed to acclimatise for at least 2 min. The mechanical stimulus was delivered at a 90° angle from above to the dorsal side of abdomen (in the region of segments 5–7). A positive response was scored if at least one 360° rotation was elicited by the stimulus. Each larva was tested only once. The von Frey filament was constructed using 0.2 mm diameter nylon monofilament fishing line attached to a cocktail stick (de Sousa et al., 2014). Filament force (mN) was calculated using a laboratory balance by measuring the mass (g) upon filament bending and multiplying by the gravitational acceleration constant (*g*; 9.8). The 12 mm filament used in this study produced a force of 49.78 mN ± 0.43 (mean ± s.e.m, *n* = 15).

### 3.3 Fly stocks

*OrR* (wild-type), *ppk-GAL4* (kind gift from Wes Grueber), *Piezo^KO^* (B#58770), *UAS-CD8-GFP*(Lee and Luo, 1999). All stocks and crosses were raised at 25 °C on standard *Drosophila* food.

### 3.4 Larval imaging

A 3rd instar larva was immobilised beneath a coverslip on a microscope slide. Coverslip bridges either side of the larva prevented crushing. GFP was imaged live using a Nikon A1R FLIM confocal microscope. Images were processed in Fiji.

### 3.5 Larval movies

Larvae were filmed through the eyepiece of a dissection microscope using an iPhone camera attached with a Solomark Universal adapter mount. Movies were edited in iMovie.

## Author contributions

All authors carried out the experimental procedures and contributed to data analysis, BD drafted the manuscript.

## Funding

This work was supported by a 10.13039/501100000848University of Edinburgh Biomedical Teaching Organisation Research Scholarship awarded to SH, a University of Edinburgh WR Henderson Scholarship awarded to YS, a 10.13039/100010269Wellcome Trust Seed Grant (09910/Z/15/Z) and 10.13039/501100000288Royal Society Research Grant (RG170159) awarded to BD.

## CRediT authorship contribution statement

**Denis Beqja:** Methodology, Validation, Investigation, Writing - review & editing. **Sunna Haidar:** Methodology, Validation, Investigation, Writing - review & editing. **Mihail Nikolaev:** Methodology, Validation, Investigation, Writing - review & editing. **Yilan Shen:** Methodology, Validation, Investigation, Writing - review & editing. **Barry Denholm:** Conceptualization, Methodology, Validation, Formal analysis, Investigation, Resources, Writing - original draft, Visualization, Supervision, Project administration, Funding acquisition.

## Declaration of Competing Interest

The authors declare that they have no known competing financial interests or personal relationships that could have appeared to influence the work reported in this paper.
